# Matrix metalloproteinase‐9 gene polymorphisms are associated with ischemic stroke severity and early neurologic deterioration in patients with atrial fibrillation

**DOI:** 10.1002/brb3.1291

**Published:** 2019-04-22

**Authors:** Xingyang Yi, Qiang Zhou, Guo Sui, Daofeng Fan, Yongyin Zhang, Minjie Shao, Zhao Han, Hua Luo, Jing Lin, Ju Zhou

**Affiliations:** ^1^ Department of Neurology People’s Hospital of Deyang City Deyang China; ^2^ Department of Neurology The Third Affiliated Hospital of Wenzhou Medical University Wenzhou China; ^3^ Nursing Department The People’s Hospital of Deyang City Deyang China; ^4^ Department of Neurology The Affiliated Longyan first Hospital of Fujian Medical University Longyan China; ^5^ Department of Neurology The Affiliated Wenling Hospital of Wenzhou Medical University Wenling China; ^6^ Department of Neurology The Second Affiliated Hospital and Yuying Children’s Hospital of Wenzhou Medical University Wenzhou China; ^7^ Department of Neurology The Affiliated Hospital of Southwest Medical University Luzhou China

**Keywords:** atrial fibrillation, early neurological deterioration, ischemic stroke, *MMP‐9* variants, stroke severity

## Abstract

**Objectives:**

The mechanisms of ischemic stroke severity and early neurologic deterioration (END) are not fully understood. The aim of the present study was to investigate the association of six variants in *MMP‐9* gene with ischemic stroke severity and the risk for END in ischemic stroke (IS) patients with atrial fibrillation (AF).

**Methods:**

This was a multi‐center, prospective, observational study of 615 acute IS patients with AF admitted to six participating hospitals between June 2016 and October 2017. Ischemic stroke severity was assessed using the National Institutes of Health Stroke Scale (NIHSS) score on admission. END was defined as an increase of four or more points in NIHSS within 10 days of admission. Six variants of *MMP‐9* gene were examined using mass spectrometry.

**Results:**

Among the 615 enrolled patients, 112 (18.2%) patients presented with moderate or severe stroke (NIHSS score ≥16), and 108 (17.6%) patients suffered from END within 10 days of admission. Multiple logistic analysis showed that prestroke antiplatelet therapy, prestroke anticoagulant therapy, rs3918242 CT/TT, and rs3787268 AG/GG were independent predictors for stroke severity. Cox proportional hazard regression revealed that diabetes mellitus, prestroke antiplatelet therapy, prestroke anticoagulant therapy, rs1056628 AC/CC, and rs3918242 CT/TT were independently associated with the risk of END.

**Conclusions:**

The incidence of moderate or severe stroke and END was very common in acute IS patients with AF. *MMP‐9* polymorphisms were independently associated with severe stroke and higher risk of END, and prestroke antithrombotic treatment was associated with less severe stroke and lower risk of END in patients with AF.

## 1 BACKGROUND

Stroke is a major cause of death and adult disability (Luengo‐Fernandez et al., 2013), and there are approximately 3–4 million new stroke cases every year in China, with roughly 80% being ischemic strokes (IS) (Guan et al., 2017; Jiang et al., 2006; Wang et al., 2007). Atrial fibrillation (AF) is an independent risk factor for IS, and accounts for 10%–25% of all ischemic strokes (Meschia et al., 2014; Sposato et al., 2015). Recanalization of occluded vessels after embolic stroke is the only therapeutic intervention available to treat acute IS (Powers et al., 2018). However, the rate of recanalization treatment is very low (Wang et al., 2007).

Initial ischemic stroke severity is associated with post‐stroke hemorrhagic transformation (HT), mortality and functional outcomes (Huang, Weng, Su, & Lin, 2018; Lee, Lee, & Jung, 2018). Compared with other stroke subtypes, IS patients with AF are known to be related to stroke severity and stroke outcomes (Park et al., 2016). Stroke severity is dependent on the type of stroke, duration of vessel occlusion, and is also influenced by several toxic mechanisms, most identified in experimental animal models of stroke (Endres et al., 2008). Brain repair involves mechanisms differentially activated in space and time, including inflammation, brain remodeling and relearning of activated neural networks (Pekna & Pekny, 2012; Wieloch & Nikolich, 2006). Genetic factors impact on the innate cellular mechanisms and environmental factors, affecting the severity of brain damage and the subsequent functional outcomes (Cramer & Procaccio, 2012).

Early neurological deterioration (END) is fairly common in patients with acute IS, and is associated with increased morbidity and mortality (Vahidy et al., 2014; Yi, Han, Zhou, Lin, & Liu, 2016). It is very important to investigate the mechanisms of END, and prevent this detrimental event. Although our previous studies have shown that *cytochrome P450 (CYP)* genetic variants, CYP metabolite levels, antiplatelet drug resistance, and dual therapy with clopidogrel and aspirin are associated with END risk (Lin et al., 2018; Yi et al., 2016; Yi, Lin, Li, Zhou, & Han, 2017; Yi, Lin, Wang, & Zhou, 2017; Yi et al., 2016), the underlying mechanisms of END remain unclear. Furthermore, previous studies did not investigate the incidence and possible mechanisms of END in acute IS patients with AF.

Matrix metalloproteinases (MMPs), well‐known inflammatory mediators, belong to a family of structurally related zinc‐binding proteolytic enzymes that are widely distributed in human tissues. In cerebral ischemia there is an enhanced expression of MMP‐9, which has been associated with various complications including excitotoxicity, neuronal damage, apoptosis, and blood–brain barrier (BBB) opening leading to cerebral edema, and HT (Candelario‐Jalil, Yang, & Rosenberg, 2009; Fatar, Stroick, Griebe, & Hennerici, 2005; Kurzepa, Kurzepa, Golab, Czerska, & Bielewicz, 2014). MMP‐9 protein is involved in inflammatory responses trigged by ischemia, and it plays a key role in BBB destruction and neuronal damage (Barr et al., 2010). MMP‐9 inhibitors can decrease the BBB destruction in the experimental stroke model (Lu et al., 2009). The *MMP‐9* gene is located on chromosome 20q12.2–13.1. Previous studies have demonstrated that MMP‐9 activity is controlled by single nucleotide polymorphisms (SNPs) of *MMP‐9* gene (Blankenberg et al., 2003). Several reports have shown that *MMP‐9* polymorphisms are associated with carotid atherosclerosis and increased IS risk (Lin et al., 2012; Yuan et al., 2013). However, there are few studies to evaluate the association of *MMP‐9* gene polymorphisms with initial ischemic stroke severity and the risk for END IS patients with AF.

With this background we aimed to investigate whether *MMP‐9* gene polymorphisms influence initial ischemic stroke severity and the risk of END in acute IS patients with AF in the Chinese population. This study was expected to provide novel insight into the mechanisms for stroke severity and END, and prevent this detrimental event.

## 2 MATERIALS AND METHODS

### 2.1 Ethics statement

This was a multi‐center, prospective, observational study, which was conducted in the People's Hospital of Deyang City, the Second Affiliated Hospital and Yuying Children's Hospital of Wenzhou Medical University, the Third Affiliated Hospital of Wenzhou Medical University, the Affiliated Wenling Hospital of Wenzhou Medical University, the Affiliated Hospital of Southwest Medical University, and the Affiliated Longyan first Hospital of Fujian Medical University between June 2016 and October 2017. The study protocol was reviewed and approved by the ethics committee of the participating hospitals. Each of the participants provided an informed consent (in Chinese language) before participating in this study.

### 2.2 Study populations

Between June 2016 and October 2017, we consecutively enrolled the first‐ever IS patients with known history of AF or atrial flutter within 48 hr of stroke onset. All enrolled patients had relevant cerebral lesions on diffusion weighted magnetic resonance imaging. AF or atrial flutter was confirmed by common electrocardiogram (ECG) or 24‐hr Holter ECG during in‐hospital. All patients underwent a baseline brain computed tomography (CT) scan and magnetic resonance (MR) imaging on admission. CT angiography (CTA) or MR angiography (MRA) of the brain and color duplex ultrasound of the carotid arteries were assessed in all patients. The inclusion criteria were acute IS (≤48 hr of stroke onset) patients with nonvalvular AF or atrial flutter. Exclusion criteria were: (a) valvular AF or AF with prosthetic heart valve; (b) previous stroke or TIA; (c) IS caused by atherothrombosis, small artery disease, other or unknown factors; (d) patients who were receiving unfractionated heparin, low‐molecular‐weight heparin within 7 days of stroke onset; (e) intravenous thrombolytic therapy or intra‐arterial catheter‐based treatment; (f) hypoxia, fever, or any relevant hemodynamic compromise on admission; (g) severe cardiovascular, liver, or renal disease; and (h) unwillingness to participate in this study. All enrolled patients received standard therapies based on standard guidelines (Kernan et al., 2014; Powers et al., 2018).

The following data were recorded: (a) age, sex, history of hypertension and diabetes mellitus, dyslipidemia, smoking, and coronary artery disease or myocardial infarction; (b) fasting total plasma cholesterol (TC), triglycerides (TG), low‐density lipoprotein cholesterol (LDL‐C), fasting glucose, and hemoglobin A1c. Dyslipidemia was defined as TC > 200 mg/dl, TG > 180 mg/dl or use of lipid‐lowering medication (Yi et al., 2016; Yi, Lin, Li, Zhou, et al., 2017); (c) prestroke medication and in‐hospital treatment. Prestroke antithrombotic treatment, including antiplatelet therapy, warfarin, or non‐vitamin K antagonist oral anticoagulants (NOACs), was defined as documentation of patients receiving an antithrombotic agent within 7 days before their index stroke onset.

### 2.3 Genotyping for MMP‐9

We selected six SNPs of *MMP‐9* gene from the NCBI database (http://www.ncbi.nlm.nih.gov/SNP), including rs3918242, rs3787268, rs1056628, rs2664517, rs17576, and rs2250889, according to the following criteria: (a) SNPs that had been assessed in previous studies (Adams et al., 1999; Blankenberg et al., 2003; Lin et al., 2012; Yuan et al., 2013); (b) tag major haplotypes with minor allele frequency >0.05 based on international HapMap data on NCBI Build 36 assembly for Asian population; (c) the SNPs lead to amino acid changes.

Whole blood (3 ml) from each patient was drawn from an arm vein into a sterile tube containing ethylenediaminetetraacetic acid and genomic DNA was extracted using a modified phenol/chloroform method and purified using a UNIQ‐10 kit (Sangon Biotech Co., Ltd., Shanghai, China). The genotyping of the six SNPs was performed using the matrix‐assisted laser desorption/ionization time of flight mass spectrometry method as previously described (Lin et al., 2018; Yi, Lin, Li, Zhou, et al., 2017; Yi et al., 2017). For each variant, subjects were dichotomized a priori into two groups based on its known effect on enzymatic function according to the literature and with the use of established common‐consensus star allele nomenclature (Lin et al., 2018; Yi et al., 2017).

### 2.4 Initial stroke severity and assessment of END

For each patient, National Institutes of Health Stroke Scale (NIHSS) assessment was performed by a member of the stroke team on admission, and subsequently on a daily basis throughout the period of hospitalization. An additional NIHSS assessment was performed whenever examination deteriorated. Upon notification of deterioration, a stroke team member reassessed the patient and performed the additional NIHSS evaluation. Initial ischemic stroke severity was assessed using NIHSS score on admission, and patients with an NIHSS score ≥ 16 were classified as having a moderate or severe stroke (Adams et al., 1999; Powers et al., 2018). According to our previous studies (Yi, Chi, Wang, Zhang, & Lin, 2015; Yi, Lin, Wang, Zhang, & Chi, 2014), END was defined as an increase of four or more points in NIHSS within 10 days of admission, while excluding a new infarct in another vascular territory or HT of infarct.

### 2.5 Statistical analysis

We speculated that a sample of 600 patients would provide 80% power to detect a relative risk increment of 10% in the percentage of END in patients carrying variant genotypes, with a two‐sided type I error of 0.05, assuming an event rate of 15% in patients carrying wild‐type genotypes.

Baseline clinical characteristics were compared using Student *t* test (continuous variables) and *χ* ^2^ test (categorical variables) between patients with NIHSS score ≥16 and <16 or between patients with and without END. Deviation of Hardy–Weinberg equilibrium for genotype frequencies was also analyzed using goodness‐of‐fit *χ* ^2^‐test (Wang et al., 2018; Zhang et al., 2018). Difference of genotype frequencies was compared using *χ* ^2^‐test between patients with NIHSS score ≥16 and <16 or between patients with and without END. Multivariable logistic regression analysis was employed to investigate independent predictors for initial stroke severity, and reported as odds ratio (OR) with 95% confidence interval (CI). Cox proportional‐hazards model was used to assess the risk factors for END, and reported as hazard ratio (HR) with 95% CI. Variables entered the models were traditional risk factors (such as age, sex, hypertension and diabetes mellitus) and the variables showed a significant association (*p* < 0.1) with stroke severity or END on univariate analysis.

All statistical analyses were performed using SPSS 16.0 (SPSS Inc., Chicago, IL). All tests were two sided, and a *p* value less than 0.05 was considered statistically significant.

## 3 RESULTS

Among the 615 enrolled patients, 112 (18.2%) patients presented with moderate or severe stroke (NIHSS score ≥16 on admission). All genotype frequencies of the six SNPs in patients were in Hardy–Weinberg equilibrium (rs3918242, *p* = 0.476; rs3787268, *p* = 0.386; rs1056628, *p* = 0.417; rs2664517, *p* = 0.198; rs17576, *p* = 0.275; rs2250889, *p* = 0.323). Univariate analyses revealed that international normalized ratio (INR) on admission, prestroke antiplatelet therapy, prestroke anticoagulant therapy, rs3918242 CT/TT, and rs3787268 AG/GG were significantly associated with moderate or severe stroke (all *p* < 0.05, Table 1). The proportion of patients presenting with moderate or severe stroke was significantly higher in patients carrying rs3918242 CT/TT than those carrying rs3918242 CC (wild‐type genotype) (31.8% [35/110] vs. 15.2% [77/505], respectively, *p* < 0.001), or patients carrying rs3787268 AG/GG than those carrying 3787268 AA (wild‐type genotype) (21.6% [79/365] vs. 13.2% [33/250], respectively, *p* = 0.009, Table 1).

**Table 1:** Characteristics and genotype distributions in patients with NIHSS score ≥16 and NIHSS score <16

Variables	NIHSS score ≥ 16 (*n* = 112)	NIHSS score < 16 (*n* = 503)	*P* value
Age (years)	69.9 ± 15.3	71.2 ± 17.1	0.442
Men (*n*, %)	62 (55.4)	288 (57.3)	0.714
Hypertension (*n*, %)	91 (81.3)	418 (83.1)	0.676
Diabetes mellitus (*n*, %)	42 (37.5)	154 (30.6)	0.183
Atrial fibrillation (*n*, %)	112 (100.0)	503 (100.0)	—
Dyslipidemia (*n*, %)	69 (61.6)	312 (62.0)	0.998
History of CAD or MI (*n*, %)	12 (10.7)	64 (12.7)	0.554
Fasting glucose (mM)	6.4 ± 2.1	6.3 ± 2.7	0.673
INR on admission	1.2 ± 0.4	1.3 ± 0.5	0.031
Prestroke CHA_2_DS_2_‐VASc score (*n*, %)			
0–1	5 (4.5)	27 (5.4)	0.692
≥2	107 (95.5)	476 (94.6)	0.692
Onset to admission time (hr)	30.8 ± 13.6	28.9 ± 14.7	0.187
Prestroke treatment (*n*, %)			
Antihypertensive	90 (80.4)	410 (81.5)	0.793
Hypoglycemic	31 (27.7)	146 (29.0)	0.794
Statins	21 (18.8)	100 (19.9)	0.798
Antiplatelet therapy	40 (35.7)	235 (46.7)	0.039
Anticoagulant therapy	10 (8.9)	117 (23.3)	<0.001
Rs1056628			
AA	74 (66.1)	340 (67.6)	0.689
AC + CC	38 (33.9)	163 (32.4)	
Rs3918242			
CC	77 (68.7)	428 (85.1)	< 0.001
CT + TT	35 (31.3)	75 (14.9)	
Rs2664517			
CC	104 (100.0)	601 (100.0)	—
Rs17576			
AA	12 (10.7)	59 (11.7)	0.688
AG + GG	100 (89.3)	444 (88.3)	
Rs3787268			
AA	33 (29.5)	217 (43.1)	0.009
AG + GG	79 (70.5)	286 (56.9)	
Rs2250889			
CC	67 (59.8)	276 (54.9)	0.323
CG + GG	45 (40.2)	227 (45.1)	

Note

NIHSS, National Institutes of Health Stroke Scale; CAD, coronary artery disease; MI, myocardial infarction; INR, international normalized ratio; CHA_2_DS_2_‐VASc score, congestive heart failure, hypertension, age ≥ 75 years (doubled), diabetes, stroke/transient ischemic attack/thromboembolism (doubled), vascular disease (priormyocardial infarction, peripheral artery disease, or aortic plaque), age 65–75 years, sex category (female).

Multiple logistic regression showed that prestroke antiplatelet therapy (OR: 0.68; 95% CI: 0.52–0.98; *p = *0.038), prestroke anticoagulant therapy (OR: 0.62; 95% CI: 0.47–0.92; *p = *0.025), rs3918242 CT/TT (OR: 2.01; 95% CI: 1.23–4.65; *p = *0.006), and rs3787268 AG/GG(OR:1.83; 95% CI: 1.12–3.67; *p = *0.022) were independent predictors for initial stroke severity (Table 2).

**Table 2:** Multivariable logistic regression analysis of independent predictors for initial stroke severity (NIHSS score ≥16)

Variables	OR	95% CI	*p* Value
Age	0.92	0.76–1.32	0.521
Diabetes mellitus	1.13	0.89–1.96	0.418
Hypertension	0.82	0.71–1.28	0.576
INR on admission	0.86	0.67–1.14	0.246
Prestroke antiplatelet therapy	0.68	0.52–0.98	0.038
Prestroke anticoagulant therapy	0.62	0.47–0.92	0.025
Rs3918242 CT/TT	2.01	1.23–4.65	0.006
Rs3787268 AG/GG	1.83	1.12–3.67	0.022

Note

OR for continuous variables means per 1‐ Standard Deviation increase.

NIHSS, National Institutes of Health Stroke Scale; OR, odds ratio; CI, confidence interval.

A total of the 615 patients, the average hospital stay was 14.8 days. There were no patients discharged within 10 days of admission. Among them, 108 (17.6%) patients suffered from END within 10 days of admission. Furthermore, 77 patients (71.3%) deteriorated within the first 48 hr of admission. The median (interquartile range) increase in total NIHSS score was 6 (4–13) at the time of deterioration.

Compared with patients without END, there was older age, the proportion of diabetes mellitus and level of fasting glucose were higher, INR on admission was lower, the percentage of prestroke antiplatelet therapy or anticoagulant therapy was lower, and the frequency of rs1056628 AC/CC or rs3918242 CT/TT was significantly higher in patients who experienced END (Table 3). Furthermore, the incidence of END was significantly higher in patients carrying 1056628 AC/CC than those carrying rs1056628 AA (wild‐type genotype) (23.4% [47/201] vs. 14.7% [61/414], respectively, *p* = 0.009) or patients carrying rs3918242 CT/TT than those carrying rs3918242 CC (wild‐type genotype) (30.0% [33/110] vs. 14.9% [75/505], respectively, *p* < 0.001, Table 3).

**Table 3:** Characteristics and genotype distributions in patients with and without END

Variables	Patients with END (*n* = 108)	Patients without END (*n* = 507)	*p* Value
Age (years)	72.9 ± 14.2	69.5 ± 16.4	0.036
Men (*n*, %)	61 (56.5)	289 (57.0)	0.998
Hypertension (*n*, %)	91 (84.3)	418 (82.4)	0.676
Diabetes mellitus (*n*, %)	47 (41.9)	149 (29.4)	0.005
Atrial fibrillation (*n*, %)	108 (100.0)	507 (100.0)	—
Dyslipidemia (*n*, %)	68 (62.9)	313 (61.7)	0.997
Systolic blood pressure (mm Hg)	155.8 ± 16.7	153.8 ± 17.7	0.233
Diastolic blood pressure (mm Hg)	91.4 ± 13.8	89.8 ± 17.5	0.241
Fasting glucose (mM)	7.4 ± 2.3	6.1 ± 2.7	<0.001
Onset to admission time (hr)	29.6 ± 14.2	30.7 ± 16.3	0.382
NIHSS score at admission	14.2 ± 3.6	14.4 ± 4.3	0.615
INR on admission	1.1 ± 0.5	1.3 ± 0.4	<0.001
Prestroke treatment (*n*, %)			
Antihypertensive	89 (82.4)	411 (81.1)	0.999
Hypoglycemic	40 (37.0)	137 (27.0)	0.041
Statins	18 (16.7)	103 (20.3)	0.401
Antiplatelet therapy	37 (34.3)	238 (46.9)	0.018
Anticoagulant therapy	9 (8.3)	118 (23.3)	<0.001
In‐hospital treatment (*n*, %)			
Antihypertensive drugs	92 (85.2)	434 (85.6)	0.999
Hypoglycemic drugs	46 (42.6)	183 (36.1)	0.208
Statins	100 (92.6)	472 (93.1)	0.997
Antiplatelet therapy	35 (32.4)	165 (32.5)	0.997
Anticoagulant therapy	44 (40.7)	203 (40.0)	0.999
Rs1056628			
AA	61 (56.5)	353 (69.6)	0.009
AC + CC	47 (43.5)	154 (30.4)	
Rs3918242			
CC	75 (69.4)	430 (84.8)	<0.001
CT + TT	33 (30.6)	77 (15.2)	
Rs2664517			
CC	104(100.0)	601 (100.0)	—
Rs17576			
AA	11 (10.2)	60 (11.8)	0.648
AG + GG	97 (89.8)	447 (88.2)	
Rs3787268			
AA	39 (36.1)	211 (41.6)	0.296
AG + GG	69 (63.9)	296 (58.4)	
Rs2250889			
CC343	63 (58.3)	280 (55.2)	0.574
CG + GG272	45 (41.7)	227 (44.8)	

Note

END, early neurologic deterioration; NIHSS, National Institutes of Health Stroke Scale; INR, international normalized ratio.

Cox proportional hazards model revealed that diabetes mellitus (HR: 1.56; 95% CI: 1.02–2.97; *p = *0.042), prestroke antiplatelet therapy (HR: 0.67; 95% CI: 0.62–0.97; *p = *0.035), prestroke anticoagulant therapy (HR: 0.65; 95% CI: 0.53–0.96; *p = *0.028), rs1056628 AC/CC (HR: 1.93; 95% CI: 1.09–4.03; *p = *0.019), and rs3918242 CT/TT (HR: 2.12; 95% CI: 1.36–5.15; *p = *0.005) were independently associated with the risk of END (Table 4).

**Table 4:** Cox proportional hazard regression analysis of independent risk factors for early neurologic deterioration

Variables	OR	95% CI	*p* Value
Age	0.89	0.81–1.37	0.428
Diabetes mellitus	1.56	1.02–2.97	0.042
Hypertension	0.82	0.78–1.26	0.484
Fasting glucose	0.99	0.98–2.35	0.137
INR on admission	0.88	0.76–1.29	0.312
Prestroke hypoglycemic therapy	0.86	0.85–1.19	0.435
Prestroke antiplatelet therapy	0.67	0.62–0.97	0.035
Prestroke anticoagulant therapy	0.65	0.53–0.96	0.028
Rs1056628 AC/CC	1.93	1.09–4.03	0.019
Rs3918242 CT/TT	2.12	1.36–5.15	0.005

Note

HR for continuous variables means per 1‐ Standard Deviation increase.

NIHSS, National Institutes of Health Stroke Scale; HR, hazard ratio; CI, confidence interval.

## 4 DISCUSSION

With this multi‐center, prospective, observational study, we investigated the possible role of *MMP‐9* gene polymorphisms in initial stroke severity and END risk in IS patients with AF. The results showed that the proportion of patients presenting with moderate or severe stroke was 18.2%, and 17.6% of patients suffered from END. Prestroke antiplatelet therapy, prestroke anticoagulant therapy, rs3918242 CT/TT, and rs3787268 AG/GG were independent predictive factors for initial stroke severity by multivariable logistic regression. Cox proportional hazards model revealed that diabetes mellitus, prestroke antiplatelet therapy, prestroke anticoagulant therapy, rs1056628 AC/CC, and rs3918242 CT/TT were independently associated with the risk of END.

Platelet activation may increase atherogenesis and promote blood vessel wall injuries, and has a crucial role in thrombus extension and ischemic damage (Fateh‐Moghadam et al., 2005). Thus, the current guideline recommends oral administration of aspirin within 48 hr of stroke onset in patients with acute IS to reduce mortality and unfavorable outcomes (Powers et al., 2018). However, the effect of prestroke aspirin use on initial stroke severity or END is controversial. One recent study demonstrated that strokes were less severe in patients already taking aspirin (Park et al., 2016), whereas some others have not shown an association between prestroke aspirin use and initial stroke severity (Kim et al., 2010). We previously reported that prestroke concomitant statin and aspirin use was associated with lower END and platelet activity in IS patients with atherothrombosis or small artery disease (Yi, Han, Wang, Zhou, & Lin, 2017). The results of our study show that prestroke antiplatelet therapy may reduce initial stroke severity and the incidence of END in IS patients with AF. Aspirin may benefit IS patients by improving the microcirculation in the ischemic penumbra through inhibition of platelet‐derived vasoconstrictors, such as thromboxane A2 (Rosenblum & El‐Sabban, 1977). In addition, aspirin may also limit clot size, extent of thrombosis, and subsequent embolism (Joseph, Han, Tsering, Grunfeld, & Welch, 1992). Furthermore, aspirin has neuroprotective and antiinflammatory effects, these may be other beneficial mechanisms of aspirin (Kuhn, Muller, Bttner, & Gerlach, 1995).

AF is a potentially treatable risk factor for IS. Numerous studies have revealed that warfarin or NOACs can reduce IS risk (Patel et al., 2011). Based on these data, the current guideline recommends warfarin or NOACs for stroke prevention in high‐risk patients with AF (Meschia et al., 2014). However, oral anticoagulants are especially underused in patients with known nonvalvular AF in China (Wang et al., 2014). Several studies have demonstrated that preceding oral anticoagulants are associated with reduced initial stroke severity on admission and reduced disability or death at discharge in IS patients with AF (O'Donnell et al., 2006; Xian et al., 2017). These results were consistent with our study. Thus, preceding antithrombotic treatment is very important for reducing ischemic stroke severity and END risk in the high‐risk patients with AF.

The most interesting finding in this study was that the variants of rs3918242 and rs3787268 in *MMP‐9* gene were independent predictors for initial stroke severity, and rs1056628 AC/CC and rs3918242 CT/TT were independently associated with higher risk of END after adjusting for covariates. To the best of our knowledge, this study is the first to investigate the associations of variants in *MMP‐9* gene with initial stroke severity and END risk in IS patients with AF. However, the pathophysiological mechanisms of these variants in *MMP‐9* gene effect on initial stroke severity and END risk remain unclear.

MMP‐9 can activate numerous proinflammatory cytokines and chemokines such as interleukin and tumor necrosis factor (Candelario‐Jalil et al., 2009; Fatar et al., 2005; Kurzepa et al., 2014), facilitate leukocytes transport across the endothelium, and it plays an important role in BBB destruction, neuronal damage and subsequent HT (Barr et al., 2010; Hill, Poddar, Thompson, Rosenberg, & Yang, 2012). MMP‐9 is also involved in the generation of free radicals that result in atherosclerotic lesions and plaque rupture in carotid atherosclerotic plaques (Heo et al., 2011). The high concentration of MMP‐9 in plasma or MMP‐9 mRNA concentration within the acute phase of IS increases the risk of HT (Montaner et al., 2001), and is a predictor of poor outcomes and mortality in patients with IS (Graham et al., 2012). *MMP‐9* gene polymorphisms encode and regulate the transcription of MMP‐9 protein, and are associated with concentration of MMP‐9 in plasma (Blankenberg et al., 2003).

Several studies have investigated the association of *MMP‐9* gene polymorphisms with carotid atherosclerosis and the risk of IS or HT (Ho et al., 2015; Lin et al., 2012; Nie, Wang, & Tang, 2014; Yuan et al., 2013; Zhang, Cao, Xu, Li, & Xu, 2015). The rs3918242 polymorphism in the *MMP‐9* promoter may reduce the rate of transcription by inhibiting protein binding, which downregulates MMP‐9 expression, and is associated with carotid atherosclerosis and increased IS risk (Lin et al., 2012). Zhang et al. evaluated the rs3918242 promoter region polymorphisms and found that the CC genotype was associated with significantly more HT compared to the CT and TT genotype, and the C‐allele was significantly higher in patients with HT compared to the T‐allele (Zhang et al., 2015). However, Nie et al. showed that the T‐allele in the promoter region rs3918242 (C‐ vs. T‐ allele) was associated with increased IS risk (Nie et al., 2014). Yuan et al. studied rs1056628 in a Chinese population and noted the dose‐dependent increased expression of the C‐allele and CC genotype in IS patients compared with controls (Yuan et al., 2013). Ho et al. showed that *MMP‐9* rs3787268 polymorphisms were associated with risk of spontaneous intracerebral hemorrhage (Ho et al., 2015). In this study, we found that the variants of rs3918242 and rs3787268 in *MMP‐9* gene were independent predictors for initial stroke severity, and rs1056628 AC/CC and rs3918242 CT/TT were independently associated with the higher risk of END after adjusting for covariates in IS patients with AF. Thus, we reason that the variants in *MMP‐9* gene could potentially provide these individuals with higher concentration of MMP‐9, result in BBB destruction, apoptosis, and neuronal damage, thereby increasing the risk of END and stroke severity in these patients. However, further studies are needed to validate our findings in the future.

Despite novel findings, several limitations were noted in this study. First, the concentration of MMP‐9 in plasma was not measured in this study, which may otherwise provide additional functional information to support our hypothesis. Second, although we genotyped multiple known functional variants of the *MMP‐9* gene, some rare functional variants were not investigated in this population. Moreover, as previously mentioned, oxygen free radical damage, inflammation reactions, MMP‐2, MMP‐3 may also play a role in BBB destruction and neuronal damage (Candelario‐Jalil et al., 2009; Fatar et al., 2005; Heo et al., 2011; Hill et al., 2012; Kurzepa et al., 2014). We did not measure the variants in oxygen free radical relevant genes, inflammation relevant genes, and *MMP‐2* and *MMP‐3* genes in this study. Thus, future studies involving a larger set of genetic variants must be conducted to evaluate the full extent of relevant genetic variants effect on stroke severity and END risk. Third, due to the limited sample size and six‐center study, our findings must be confirmed with larger, multi‐center studies. Finally, the main aim of this study was to investigate the association of *MMP‐9* gene polymorphisms with initial stroke severity and the risk of END in IS patients with AF. The other stroke subtypes were excluded in this study. Thus, we did not know whether there was an effect of *MMP‐9* gene polymorphisms on stroke severity and END risk in other stroke subtypes.

## 5 CONCLUSION

The incidence of moderate or severe stroke and END was very common in acute IS patients with AF. *MMP‐9* polymorphisms were independently associated with severe stroke and higher risk of END, and prestroke antithrombotic treatment was associated with less severe stroke and lower risk of END in patients with AF. Our findings may be useful to insight into the complex pathogenesis of stroke severity and END, prevent END, and decrease stroke severity in acute IS patients with AF. However, further studies are needed to confirm our findings in the future.

## CONFLICT OF INTEREST

The authors declare that they have no competing interest.

## Data Availability

The data that support the findings of this study are available on request from the corresponding author. The data are not publicly available due to privacy or ethical restrictions.
